# Coalescent models for developmental biology and the spatio-temporal dynamics of growing tissues

**DOI:** 10.1098/rsif.2016.0112

**Published:** 2016-04

**Authors:** Patrick Smadbeck, Michael P. H. Stumpf

**Affiliations:** Centre for Integrative Systems Biology, Imperial College London, London SW7 2AZ, UK

**Keywords:** agent-based modelling, coalescent theory, lineage tracing, developmental biology, tissue growth models

## Abstract

Development is a process that needs to be tightly coordinated in both space and time. Cell tracking and lineage tracing have become important experimental techniques in developmental biology and allow us to map the fate of cells and their progeny. A generic feature of developing and homeostatic tissues that these analyses have revealed is that relatively few cells give rise to the bulk of the cells in a tissue; the lineages of most cells come to an end quickly. Computational and theoretical biologists/physicists have, in response, developed a range of modelling approaches, most notably agent-based modelling. These models seem to capture features observed in experiments, but can also become computationally expensive. Here, we develop complementary genealogical models of tissue development that trace the ancestry of cells in a tissue back to their most recent common ancestors. We show that with both bounded and unbounded growth simple, but universal scaling relationships allow us to connect coalescent theory with the fractal growth models extensively used in developmental biology. Using our genealogical perspective, it is possible to study bulk statistical properties of the processes that give rise to tissues of cells, without the need for large-scale simulations.

## 1. Introduction

The connection between space and time is fundamental to developmental biology. For over a century, the location of stem cell proliferation and differentiation during development has been known to be well organized and of paramount importance to cell fate decision-making (e.g. Spemann organizer and primitive knots) [1]. Through the control of cell division and other cellular actions, spatio-temporal chemical signalling forms complex patterning vital to proper tissue development [2,3]. Despite the long-established importance of spatial information in understanding tissue development, it was not until relatively recently that widespread understanding of these effects has become possible.

More recent experimental work (relying on advanced microscopy [4] with suitable dyes [5] and fluorescence tags [6], etc.) in the context of developmental biology has focused on cell tracking and lineage tracing. These experiments have already given rise to profound new insights. Opacity, three-dimensional effects and stochasticity all make lineage tracing and cell-tracking experiments difficult [5,7], however. Even if supported by state-of-the-art computational and statistical analyses, these experiments will remain challenging. Computational modelling is therefore emerging as a desirable, and ultimately essential, tool to understand the carefully orchestrated processes underlying tissue growth and homeostasis. Mathematical or computational models can encapsulate complicated and quantitative mechanistic hypotheses and be used to test systematically which aspects of these hypotheses are borne out by reality.

In tissue and tumour modelling, agent-based models (ABMs) are gaining in popularity [8] and allow the inclusion of cellular composition of tissues from the outset. Like cells, agents interact with their environment and each other; occupy finite spatial areas/volumes; and can exhibit the hallmarks of cell behaviour: differentiation, proliferation, movement and death [9–11]. All of these factors lead to cells organizing themselves into tissues. While there is a large deterministic component underlying tissue growth (as well as homeostasis), experiments tracing cells and their progeny often demonstrate substantial variability in the lineage behaviours [12–14] easily captured by ABMs. Thus, ABM approaches provide a natural computational complement to lineage-tracing experiments.

We draw inspiration from recent modelling approaches to tissue growth and development [15] that exhibit dominant ancestral lineages called *superstars*. ABMs that describe neural crest growth and development suggest that the competition between cells for space appears to affect the development of the enteric nervous system. As cells produce offspring differences emerge in the number of progeny produced, resulting in progeny from one or very few ancestral cells dominating the tissue (or section of tissue). Similar emergent phenomena are reported from other lineage-tracing studies in both healthy and malignant tissue growth [16–18].

The most glaring issue is that of computational load. The realization of many biological functions involves billions of interacting cells. Simulating a single example of such a system may take hundreds of central processing unit hours. If we want to use statistical methods to calibrate such models against data, then we would require hundreds or thousands of such simulations, which can be all but impossible to implement without incurring massive computational cost [11]. Fortunately, by implementing methodologies inspired by population genetics, it may be possible to distil many important lineage-tracing concepts into easily digestible and computationally light quantitative rules instead.

When we trace the ancestral relationships among the cells in a tissue, we recover genealogical relationships that are familiar from population genetics. Population genetics has been applied with great success to, for example, map out the genetic history of human and animal populations, estimate the age of alleles and map out past population movements [19]. A sophisticated mathematical framework has been developed that allows us to elucidate evolutionary dynamics; for example, in a population of *N* alleles that evolve according to the standard neutral model, the average time until an allele becomes fixed is 2*N* generations [19]. One of the reasons for the success of population genetic theory is that evolutionary processes typically occur over time scales that are so long that they cannot be observed experimentally. Instead, mathematical models are used to capture the evolutionary dynamics and relate them to the observed data using statistical methods—it is probably no coincidence that evolutionary theory has been linked in lock-step to developments in statistical theory and practice.

One of the fundamental insights that has made this connection between evolutionary/population genetic theory and statistics even tighter is the realization that we can reconstruct the genealogical processes underlying a sample of alleles (drawn from a large population), i.e. we do not have to model the evolution of a large population of *N* individuals forward in time, but can instead look at the stochastic process that describes the ancestral relationship among *n* (typically 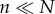) individuals/alleles [12]. Starting from the present sample, we follow their ancestral lineages back in time until all lineages have coalesced into a single lineage; this allele/state is called the most recent common ancestor (MRCA). In addition to the computational efficiency (compared with forward simulations), this *coalescent approach* also focuses explicitly on the observed data and the properties of the underlying genealogical process, and not on the lineages that result in ‘dead ends', i.e. that do not contribute to the growth front.

Here, we adapt and apply coalescent theory to developmental processes. Tissues do have MRCAs separate from the MRCA of an organism, which is, of course, the fertilized egg cell from which it originated. For most tissues, and this includes tumours, we can start from an existing tissue and go back in time until we reach a generation in which a single cell exists from which all extant cell lineages are derived. Coalescent theory allows us to study populations of cells and their ancestral relationships backwards in time and space. In fact, it is the relationship between space and time that comes to the fore in this framework.

## 2. Methods

Below, we will establish a relationship between the exact coalescent [20] (related to the Wright–Fisher model [21] of population genetics) and tissue growth models that are inspired by or are related to the classical Eden model [22] (or more general models such as the processes described by the Kardar–Parisi–Zhang (KPZ) equation [23]). To the best of our knowledge coalescent theory, ABM and fractal growth theory have not before been considered in combination in order to understand developmental processes.

### 2.1. Coalescent process

The coalescent process is a description of population evolution and underlies much of modern population genetics. The Wright–Fisher model [21] is arguably the simplest description of evolutionary change in a population of identical individuals. At each time step (where time is scaled by a generation length and measured in real-valued generations), the population (size *N*) is replaced by choosing random members of the current population to reproduce to form the next generation. The obvious advantage of such a model is its straightforward probabilistic description whereby each member of the population is equally likely to be the parent of any child in the next generation (neutral evolution).

Population genetics, however, generally seeks to obtain information about a population's history based solely on a current population's genetic data. Coalescent theory reverses time and explores a population's history by tracking how distinct lineages (branches on a family tree) eventually combine (coalesce) as the population is traced backwards in time. Kingman, in 1982 [24] (Griffiths [25] and Tajima [26] published their near-identical approaches almost simultaneously), was able to use the Wright–Fisher model and reverse time to develop this coalescent approach mathematically.

Coalescent theory [19] reverses time—the present is *t* = 0—and the number of relevant lineages (*n*) that are ancestral to the present-day sample are tracked backwards until the MRCA has been reached. These lineages coalesce (when two lineages arrive at their most-recent common ancestor), and the time spent with a specific number (*k*) of active lineages (*T_k_*) is modelled probabilistically. The key to the use of coalescent theory in population genetic analysis is in its limiting behaviour. At several common limits (large population, *N* → ∞, and small initial lineage number, 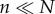), the coalescent process is characterized by an exponentially distributed reaction process, where the time before the next coalescent event among *k* lineages, *T_k_*, is given by2.1
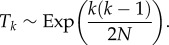
Overall, starting at a full population (*n* = *N*), the average total time to the most recent common ancestor (*T*_MRCA_) is (adapted from [19], p. 26, equation (1.31))2.2
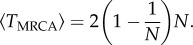
Coalescent theory has also been extended [19,27–30] to account for many features of growing populations including nonlinear growth rates (used in the tumour growth model below), where equations (2.1) and (2.2) do not apply.

While the Kingman coalescent uses this large population approximation (and in particular 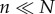) an exact description of the Wright–Fisher model is provided by Fu [20]. The fundamental difference between the two is that in conventional coalescence no more than one coalescent event can occur in any generation. The exact or 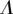-coalescent provides an exact description of the genealogy of a population (rather than a small sample) where multiple coalescent events can occur in a single time step.

### 2.2. Growth models

In total, the four models we use are all motivated by the ABM introduced by Cheeseman *et al.* [15], which corresponds to an Eden growth process [22] with diffusion. The basic assumptions are that the growth occurs at the boundary of the growing system, and that the system remains connected. The boundary is defined as cells abutting unoccupied sites (these are the only cells allowed to undergo growth in these models). In the systems presented, the boundary is predominantly composed of the leading edge of the growing tissue with very little growth occurring within the body of the tissue.

**Figure 1.**
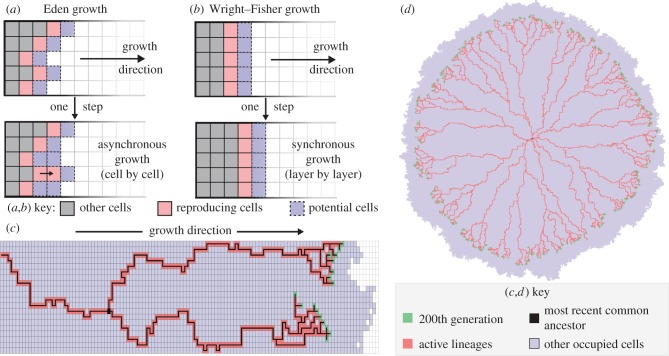
Example of Wright–Fisher and Eden results for different geometries. (*a*) A graphical description of a single step in a small Eden model. Parents are chosen uniformly at random from the neighbouring reproducing sites. (*b*) A graphical description of the Wright–Fisher growth process. Here, the reproducing cells are chosen at random to be the parent of a cell in the next generation. (*c*) An example of an Eden growth process on a bounded domain growing in a single (left-to-right) direction. The 200th generation is marked in green, active lineages in red and the MRCA in black. The MRCA is 104 generations in the past (contained within the 96th generation). (*d*) A tumour growth model (unbounded, with an initial population of one located at the origin). The 200th generation is marked in green, active lineages in red and here the MRCA is the initial generation shown in black. Star-like genealogical trees are characteristic of unbounded tissue or bacterial colony growth.

In figure 1*a*, we provide a graphical representation of a single step during Eden growth. Figure 1*b* shows the same single step for the Wright–Fisher model for comparison. In figure 1*c*, an example of lineage tracing (and coalescence) in the Eden model (*N* = 20) is shown starting at the 200th generation and tracked backwards from parent to child. The MRCA is marked in black. The time to the *T*_MRCA_ is thus measured in generations backwards in time. Figure 1*d* shows similar results for unbounded growth (e.g. in tumour growth [31]).

We use both the simple Eden growth model and the Eden growth model modified by incorporating diffusion that allows cells to move as well as reproduce; this alternative is referred to as an Eden diffusion model. In all cases, we apply periodic boundary conditions, and all simulations are conducted on a square lattice. Although this introduces a clear anisotropy in the bacterial colony structure, the critical exponents and thus the results herein are generally independent of the lattice microstructure [32,33]. Note that in both fractal growth models the simulation was stopped after a specified number of generations had been reached. More detailed descriptions of the models used in this study are provided in the electronic supplemental material.

## 3. Results and discussion

The intention of our analysis is fourfold: (i) to demonstrate the direct connection between the space and time dimensions in tissue growth; (ii) to establish that dominant lineages are a natural feature of fractal growth models that is readily captured by a coalescent process; (iii) to determine the scaling factors for coalescent models of biological growth processes and to establish the link to the classical coalescent (as applied to the Wright–Fisher model); and (iv) to show that simple scaling relationships apply to lineage tracing in both unidirectional and unbounded fractal growth systems. In terms of developmental biology, this provides a framework in which to analyse lineage-tracing experiments, as well as a means to infer properties of the ancestral (e.g. stem cell or progenitor) population; such an ability will, we hope, stimulate new experimental analyses.

### 3.1. The neutral evolution model results

**Figure 2.**
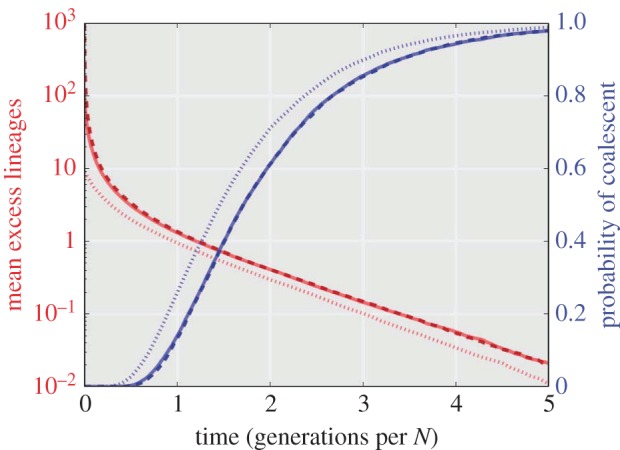
Coalescent properties for layer-by-layer tissue growth. A bounded unidirectional layer-by-layer growth process is shown for three different tissue widths (with *N* = 10 (dots), *N* = 100 (solid) and *N* = 1000 (dashed) cells). The mean excess lineages (defined as the number of lineages in addition to the single common ancestor of the population) are shown in red on a semi-log scale. The probability of having achieved coalescences by a specific cell depth/generation is shown in blue. The time axis is scaled with tissue width (*N*), revealing an asymptotic relationship as *N* → ∞. All simulations are the result of 10 000 Monte Carlo simulations.

We begin with a model whereby tissue growth proceeds in a layer-by-layer fashion with parent cells chosen at random from among the *N* cells in a population defined by the cells of the previous layer (mimicking the behaviour of the Wright–Fisher model). Such a system could be thought of as a simplified version of unidirectional and bounded (constant number of cells in each tissue layer) tissue growth. In figure 2, we show the coalescent results for three neutral evolution models with tissue widths of *N* = 10 (dots), 100 (solid), 1000 (dashes). The mean number of lineages (red) demonstrates an initial fast decay as a substantial portion of lineages are eliminated in the first few time steps. This behaviour is characteristic of the exact coalescent where many multi-lineage coalescent events occur per generation. The probability of coalescence (blue) shows that nearly all simulations will coalesce within 5 × *N* generations. By scaling the time axis with the tissue width *N*, it is easy to see how as *N* tends to infinity the coalescent process converges onto a single general trajectory characterized by equations (2.1) and (2.2). In such a simplified spatio-temporal model, the connection between tissue depth (spatial) and the generation number (temporal) is deterministic.

### 3.2. The diffusive-Eden model results

**Figure 3.**
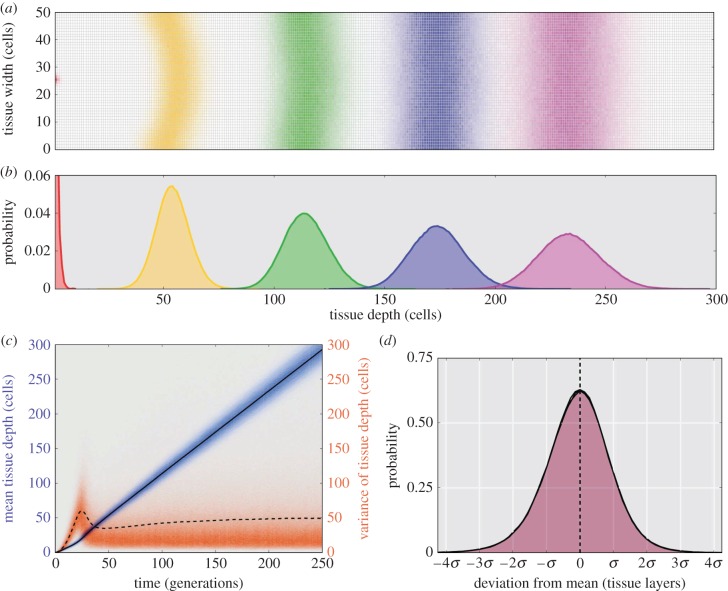
Spatio-temporal connections in the diffusive-Eden model. (*a*) Probabilistic representation of the position for generation 0 (red), 50 (yellow), 100 (green), 150 (blue) and 200 (magenta) in 10^6^ Monte Carlo realizations of the diffusive-Eden growth model with tissue width (*N*) of 50 cells. Each lattice point is weighted according to the probability that a cell of the specified generation would occupy that site during simulation. Note that the curvature of the distributions results from initializing the system as a single point. (*b*) The marginal probability distributions (summing across the tissue width) for site occupancy across the tissue depth. (*c*) Mean tissue depth (blue distribution) and the variance for the tissue depth (orange distribution) versus time as represented by the cellular generation number. The overall mean (solid line) and mean variance (dashed line) for 10^6^ simulations is also shown. Note that the variance is a long-tail distribution and thus the mean is significantly higher than expected given the distribution. (*d*) The deviation from the mean tissue depth for generations 250 (red), 200 (blue, obscured) and 150 (green, obscured) calculated from 10^6^ simulations. Note the distribution appears normally distributed with variance equal to *N* (tissue width, 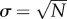) and stationary.

Figure 3*a* shows that the more realistic diffusive-Eden growth process exhibits a distinct non-deterministic spatio-temporal connection. Generations 1 (red), 50 (yellow), 100 (green), 150 (blue) and 200 (magenta) are shown with each lattice location weighted according to the probability a cell was present in 1 million stochastic realizations of the diffusive-Eden growth model. While the distribution along the tissue width quickly reaches uniformity, figure 3*b* shows how the distribution along the tissue depth appears normally distributed with an increasing mean and variance.

It should be noted that throughout this study time is represented in terms of successive generations in order to more easily translate between the neutral evolution and Eden growth models. For a particular lineage, the time since the start of the simulation is proportional to how many division events occur, and so generation number and time are interchangeable.

The increasing variance is a function of the mean tissue depth for a particular cellular generation as shown in figure 3*c*. Here, the mean tissue depth versus generation number (blue distribution, solid black line shows the mean) exhibits an increasing mean and variance. Alternatively, the variance around this mean (orange long-tailed distribution, dashed black line showing the mean value) quickly reaches a steady state. The values were calculated by taking the *N*_*g*_ cells in generation *g* in any particular simulation and obtaining a mean and variance for the tissue depth for this group. This shows an easily defined statistical relationship between time (generation number) and space (tissue depth) and thus, if coalescence in time can be determined, the statistics for spatial cell lineage tracing would be readily determined. Studies concerning related models [34] have shown a similar linear relationship between space and time in fractal growth models.

Figure 3*d* shows that there is a steady-state distribution for the deviation from the mean position for any cell (of a specific generation). The distributions are normally distributed around zero with a variance equal to *N* and very quickly reach a stationary distribution. The values were calculated by taking the *N*_*g*_ cells in generation *g* in any particular simulation and obtaining the deviations from the mean tissue depth for the group. This relationship is related to the underlying fractal growth dynamics for the Eden model whereby the roughness of a unidirectional Eden growth frontier is proportional to *N*^1/2^ [35], where *N* is the tissue width.

**Figure 4.**
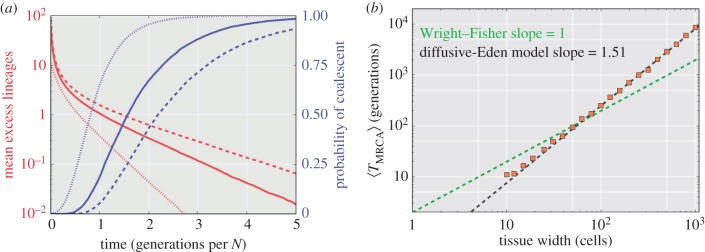
Coalescent analysis of the diffusive-Eden model with time to the most recent common ancestor scaling. (*a*) The mean excess lineages (defined as the number of lineages in addition to the single common ancestor of the population, red) and the probability of coalescence (blue). Results are shown for three tissue widths for a diffusive-Eden model, *N* = 10 (dots), 50 (solid line) and 100 (dashed line) from 10 000 Monte Carlo simulations. (*b*) The mean *T*_MRCA_ for the diffusive-Eden model is shown for tissue widths from *N* = 3 to 1000 (red squares, 1000 instances simulated) on a log–log plot. A comparison line for the Kingman coalescent (based on equation (2.2)) is shown as a green dashed line for reference. A linear regression to the diffusive-Eden results is shown as a dashed black line and exhibits a calculated slope of approximately 1.51. These linear results are used as a basis for the effective population in equation (3.1).

The next question concerns whether it is possible to apply the coalescent from the simpler neutral evolution model to the more complicated fractal growth models. In figure 4*a*, the lineage numbers (red) and probability of coalescent (blue) are shown for tissue widths of *N* = 10 (dotted line), 50 (solid line) and 100 (dashed line).

These results show two important characteristics for the diffusive-Eden model: first, the coalescence is indeed inevitable for the diffusive-Eden model with small tissue widths (e.g. *N* = 50); however, in the limit (*N* → ∞), this will not be the case. As *N* increases, the time to coalescence will drift further into the (relative) past until coalescence will not be found under reasonable time/tissue depth expectations. This suggests that, while single dominant lineages may be important on developmental spatial scales, larger tissues will be founded by several distinct ancestors derived from more general stem cells. The coalescent framework allows us therefore to estimate the number of founding stem cells of a developing tissue given its size and age.

The results from figure 4*a* beg the question as to what the scaling properties (with respect to the size of the tissue) of the 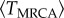 are for the diffusive-Eden model. In figure 4*b*, a log–log plot for the average *T*_MRCA_ for tissue widths from *N* = 3 to 1000 is shown (red squares, 1000 simulations). The mean time to the most recent common ancestor (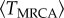, equation (2.2)) for the classical coalescent scales with *N* (producing the stationary trajectory on the normalized scale in figure 2), and is provided for comparison (dashed green line). As is apparent the slopes of the two lines are very different. A linear regression (black dashed line) can be matched to the diffusive-Eden model and shows that the slope is approximately 1.51. It has been postulated that this scaling factor is related to the dynamic exponent [36]. Indeed, this model is governed by the (1 + 1)-dimensional KPZ equation [23] that has a scaling factor of *z* = 3/2.

**Figure 5.**
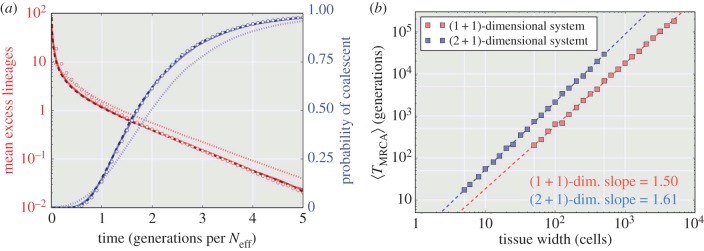
Scaled coalescent analysis for the diffusive-Eden model results. (*a*) These results are identical to those presented in figure 4*a*, but scaled with an effective population dictated by the results in figure 4*b* (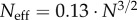, equation (3.1)). Again, *N* = 10 (dots), 50 (solid line) and 100 (dashed line) are presented. The Wright–Fisher result for *N* = 1000 (open circles) from figure 2 is provided for reference for both mean excess lineage (blue) and coalescent probability (red). (*b*) Eden model results for both (1 + 1)-dimensional and (2 + 1)-dimensional bounded growth (100 simulations per point). The slopes, as approximated by a linear regression, match the dynamic exponent for the respective (1 + 1) and (2 + 1) KPZ equation. Note that the base for the (2 + 1)-dimensional system is a square and so when the width is reported as 500 cells it means there are 250 000 cells per layer.

In figure 5*a*, the time domain for the lineage results (blue) and probability of coalescence (red) from figure 4*a* are rescaled using the *N*^3/2^ factor determined in figure 4*b*. These results now match the exact coalescent trajectory on average, and the coalescent probability becomes nearly identical with increasing *N*. As with many empirical applications of coalescent theory [37], this can be thought of as an effective population number. By scaling by the following effective population:3.1
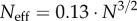

we are able to obtain an effective coalescent representation of a more realistic biological growth model for any desired tissue width (*N*). With this in mind, we can apply coalescent theory to model the ancestral relationships among cells in developing tissues.

Figure 5*b* tests the dynamic exponent scaling factor by extending the results from figure 4*b* to a (2 + 1)-dimensional system. Simulating across three orders of magnitude of tissue width (100 trajectories per simulation) the (1 + 1)- and (2 + 1)-dimensional, bounded Eden growth models exhibit a scaling factor of 1.5 and 1.61, respectively, for the mean time to the MRCA. These models of tissue development are in the same *universality class* [38] as models governed by the KPZ equation. These slopes are identical to the reported dynamic exponent for the (1 + 1)- and (2 + 1)-dimensional systems governed by the KPZ equation [36]. Using the dynamic exponent, it is then possible to determine nonlinear effective population sizes for fractal growth models.

### 3.3. Tumour Eden growth simulation

**Figure 6.**
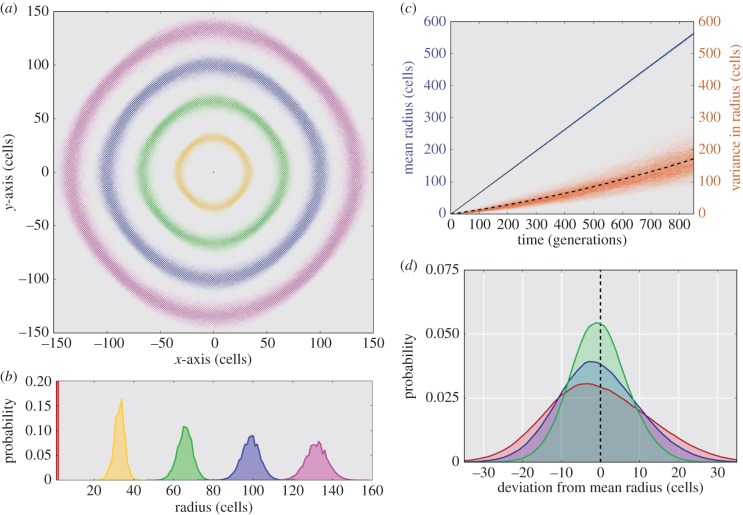
Spatio-temporal connections in a bacterial colony Eden-growth model. (*a*) Probabilistic representation of the position for generation 0 (red), 50 (yellow), 100 (green), 150 (blue) and 200 (magenta) in 10^6^ Monte Carlo realizations of the non-diffusive-Eden growth model. Each lattice point is weighted according to the probability that a cell of the specified generation would occupy that site during simulation. (*b*) A one-dimensional representation of the probability distributions for site occupancy across the radial depth. (*c*) Mean radial depth (blue distribution) and the variance for the radius (orange distribution) versus time as represented by the cellular generation number. The overall mean (solid line) and mean variance (dashed line) for 10^6^ simulations is also shown. (*d*) The deviation from the mean radius for generations 350 (green), 600 (blue) and 850 (red) calculated from 10^6^ simulations.

We next model a two-dimensional tumour or bacterial colony using a non-diffusive-Eden model without boundary constraints (see electronic supplementary material) starting from a single founder cell at the origin. Figure 6*a* shows the occupation probability of different sites for generations 1, 50, 100, 150 and 200. Once again, the relationship between time and space is apparent; see figure 6*b*.

In this case, the mean position versus generation number (figure 6*c*) is notably different from figure 3*c*. The mean radial position is almost deterministic (blue distribution and solid black line), whereas the increasing spread in the generational position data (figure 6*b*) can almost entirely be attributed to an increasing deviation from the mean. These results suggest that any individual realization of this stochastic growth process will have a non-stationary distribution as cells in the same generation move further and further apart.

In figure 6*d*, the distributions for generations 350 (green), 600 (blue) and 850 (red) are shown to diverge as the generation number increases. The lack of a stationary distribution, and thus an ever-increasing roughness to the colony surface, is a well-known consequence of unbounded Eden fractal growth [39]. It also has interesting consequences in that the MRCA is at or close to the originating cell in the system. The number of cells increases linearly with the generation number, and with non-constant growth processes the genealogical tree takes on a star-like pattern (observed experimentally in tumour growth models [31,40]).

**Figure 7.**
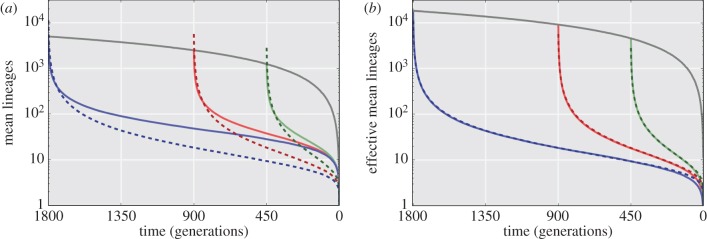
Lineages remaining starting from three generations backwards in time for a bacterial colony or tumour. (*a*) The Eden growth model for bacterial colony or tumour starting at generation 1800 (blue solid line), 900 (red solid line) and 450 (green solid line) shows the total lineages remaining. The total population (grey solid line) is growing linearly. Coalescence is not observed in almost all simulations (a star-like tree). A neutral evolution model with linear population growth (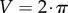) is shown as dashed lines for comparison. This model also exhibits star-like trees. (*b*) Modelling of an effective lineage number remaining now shows strong overlap between the two models. The relationship is presented in the text. All results are calculated from 1000 stochastic simulations.

In figure 7*a*, the number of ancestral lineages is traced back in time starting from generation 450 (solid green line, with *N*_0_ ≈ 1250), generation 900 (solid red line, with *N*_0_ ≈ 2500) and generation 1800 (solid blue line, with *N*_0_ ≈ 5000) of the Eden growth model. We observe that a substantial percentage of lineages remain when coalescence is forced by the linearly decreasing total population size (grey line). For comparison, classical coalescent models run for the same number of generations and an average population growth rate of 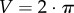 cells per generation are provided (dashed lines). Here, too, no complete coalescence occurs. However, while the two lines differ substantially, the dynamics of the tumour growth model and coalescent with linear population growth do appear to have the same basic behaviour.

The relationship between the classical coalescent model and the Eden model results can be established using the same fractal exponents observed in the diffusive-Eden model results. In 1996, Manna & Dhar [41] explored the relationship between the critical exponents of the Eden model and the underlying lineage ‘backbone’ (i.e. its genealogical tree). The key relationship concerns the fractional number of lineages that survive up to a height *h* away from the original surface, *N*_h_,3.2
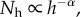
where *α*(*d*−1)/*z*, *d* is the dimension of the system (here, we are on a two-dimensional surface), and *z* is the dynamic exponent, which for the KPZ system is 3/2.

What we are actually interested in in figure 7 is the absolute (and not the fractional) number of extant lineages. Thus, we write3.3
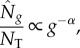
where 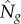 is the absolute number of lineages left *g* generations back in time and *N*_T_ is the total lineages at generation *G* – *g,* where *G* is the total number of generations simulated. *N*_T_ is proportional to *G* – *g* and thus the absolute number of lineages for the Eden system can be written as3.4
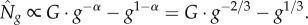


In figure 7*b*, we relate the Eden results to neutral evolution on a similarly growing population (growing linearly with generation number). Using the above graphical and numerical arguments, this neutral evolution model then scales with *α* equal to 1 and, thus,3.5
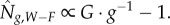


In order to translate the trajectory of the Eden growth model (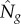) to that of the neutral evolution model (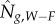) an effective lineage population for the Eden process can be formed3.6
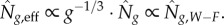


Indeed, in figure 7*b*, this simple modification of the Eden growth trajectory results in a nearly perfect overlap with the coalescent model with linear growth. The actual modification is3.7
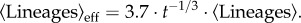


Here, time represents the generation number (starting at the first generation in the past).

Ultimately, the results for the unbounded Eden growth model agree with the results determined for the bounded growth model (and the corresponding coalescent results for growing populations found in figure 7). These results also confirm the close relationship between the dynamic exponent in fractal growth and the underlying tree structure that is the objective in cell lineage-tracing experiments. Both results suggest that it is possible to use coalescent theory to capture and describe the results found in complex fractal growth models in a computationally efficient manner. Statistically valid results about the spatial locations of cells belonging to ancestral populations of cells can also be determined from such analyses.

## 4. Conclusion

In this study, we used ABMs to simulate tissue growth and to establish shared properties of their respective ancestral trees by borrowing from the theory of coalescent processes. The resulting relationships, exemplified by shared dynamic exponents from fractal growth theory, establish non-trivial relationships between coalescent theory and fractal surface growth models relevant to developmental biology and tissue growth modelling.

The scaling property for 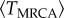 in our diffusive-Eden model is related to the dynamic exponent for Eden growth (*z* = 3/2). Indeed, research in the field of directed polymers suggests that the mean time to the MRCA will scale according to *N^z^* (written as *N*^1/*ν*^ in [36]), and it is known that the directed polymer dynamics at zero temperature are equivalent to Eden growth [42]. Thus, knowing the dynamic exponent (*z*) provides a general scaling rule that can be applied *a priori* to a biological system exhibiting fractal growth. To this end, dynamic exponents have already been experimentally determined for bacterial colonies [43]. More importantly, in our opinion, it confirms that the treatment of space as equivalent to (developmental) time is meaningful. Even in a more realistic growth model in which cells can move and rearrange, the genetic tree exhibits the same general scale for both the spatial and temporal domain.

Coalescent theory then is immediately applicable and valuable to the analysis of biological tissue and growth models, especially if the cellular nature is explicitly modelled. The emergence of dominant lineages, for example, is readily understood without the need for simulations, and can further be rationalized using the scaling properties outlined above. Additionally, the presence of a single dominant lineage for large domain sizes can now be completely ruled out for the diffusive-Eden model, because as *N* goes to infinity an MRCA will never be observed for any practical tissue depth. Going even further the presence of a dominant lineage can now potentially be ruled out for a range of tissue models, because any fractal growth system with a dynamic exponent larger than 1 is not likely to have a single MRCA within relevant tissue depths as *N* increases. Finally, we can begin to make non-trivial biological statements about systems that are too large or complicated to simulate.

Importantly, many of the results presented here can be confirmed experimentally. In particular, the determination of a dynamic exponent for an actual growing bacterial population combined with lineage-tracing experiments can confirm the results presented in figure 5. Of particular appeal is the potential to estimate the number of stem cells required to generate the cells in a tissue of given size over a certain limited time frame. We hope that this study motivates experimental analyses that allow us to gauge the required size of the stem cell pool, as this (i) would be the most stringent test for our theoretical analysis and (ii) could have profound implications for developmental biology as well as regenerative medicine. Finally, the fact that tissue growth models fall into the same universality class as KPZ models should enable some general insights into the dynamics at the surface of growing tumours, including the roughness of such tumours.

The perspective taken here focuses solely on the ancestry of cells—but so does lineage tracing. Intra- and intercellular processes that shape the decisions of cells [44,45] will be an obvious extension to consider, especially in the context of multi-scale models [46] applied to developmental processes. Already, however, this analysis provides a useful complementary framework for the analysis of lineage-tracing studies. There is, we believe, an intrinsic appeal of applying evolutionary concepts to developmental problems. Evolution does, of course, provide a framework against which we view development, but here it can also provide powerful computational tools for the analysis of tissue dynamics during growth as well as homeostasis.

## Supplementary Material

Model Descriptions and Video Captions
