# Supplementary material for: Coalescent models for developmental biology and the spatio-temporal dynamics of growing tissues
Source: J R Soc Interface. 2016 Apr;13(117):20160112. doi: 10.1098/rsif.2016.0112 (PMC4874433; doi:10.1098/rsif.2016.0112)
Supplement: Model Descriptions and Video Captions [file rsif20160112supp1.pdf]

Supplement

Growth Model Descriptions

In Eden growth models we are looking at tissues growing over time — it is for these processes that we seek genealogical representations of the ancestry of cells. We are motivated by an agent-based model introduced by Cheeseman et al. [1], which corresponds to an Eden growth process [2] with diffusion. The basic assumptions are that the growth occurs at the boundary of the growing system, and that the system remains connected. The boundary, here, is simply defined as cells abutting unoccupied sites, as a cell is only allowed to grow if it is touching an unoccupied site. In the systems presented below the boundary is predominantly comprised of the leading edge of the growing tissue with very little growth occurring within the body of the tissue. The basic algorithmic growth process (variations exist, see [3]) can be described by Algorithm 1,

Algorithm 1 Eden model

```
Initialize set of N_0 cells A, N_T = N_0 on the lattice
Determine set of boundary sites, B, from A
while N_T ≤ N_max do
    choose site b from boundary sites B
    add cell b to set A
    recalculate set of accessible boundary sites B from A
    N_T = N_T + 1
end while
```

Eden growth is a generic yet qualitatively realistic description of many growing systems in nature (including cellular systems) and it has been used extensively across many different fields [4–6]. The analysis of fractal growth systems from a coalescent perspective could bridge the gap between the simplicity of the coalescent and the complexity of realistic biological systems. In fractal growth systems cells compete for space, which is a limited resource. This limitation and asymmetric growth (cellular generations overlap) are two fundamental differences between the Wright-Fisher model and fractal growth models that complicate the use of coalescent theory in spatial lineage tracing studies.

The first fractal growth model we develop to describe tissue growth differs from Eden growth by incorporating diffusion which also allows cells to move. This model is described in Algorithm 2 and uses periodic boundary conditions in the non-growing dimension. The non-diffusive version of this model is a true Eden growth model and is used in the tumour/bacterial colony growth simulations (Algorithm 3). Note that in both fractal growth models the simulation was stopped after a specified number of generations had been reached, and all simulations are conducted on a square lattice. Although this introduces a clear anisotropy in the bacterial colony structure, the critical exponents and thus the results herein are generally independent of the lattice microstructure [7, 8].

In total four distinct models are used to simulate tissue growth in this study:

- Kingman Model - non-spatial system growing synchronoously, Figure 2.

Algorithm 2 Diffusive-Eden Model

```
Initialize set of N_0 cells A, g = 1
while g ≤ g_max do
    choose cell a from current cell population A to diffuse
    choose an adjacent site (d) for diffusion
    if d is unoccupied then
        move cell a to site d
    end if
    choose cell a from current cell population A to proliferate
    g_a is the generation number for the cell a
    choose an adjacent site (p) for proliferation
    if p is unoccupied then
        generate new cell at site p, add to set A
        the generation number for the cell p is g_p = g_a + 1
        if g_p > g then
            g = g_p
        end if
    end if
end while
```

Algorithm 3 Eden Model for Colony Growth

```
Initialize set of N_0 cells A, g = 1
while g ≤ g_max do
    choose cell a from current cell population A to proliferate
    g_a is the generation number for the cell a
    choose an adjacent site (p) for proliferation
    if p is unoccupied then
        generate new cell at site p, add to set A
        the generation number for the cell p is g_p = g_a + 1
        if g_p > g then
            g = g_p
        end if
    end if
end while
```

- Eden Model (1+1 dimensions) - Two-dimensional system growing as a one dimensional surface. Figure 1, Figure 5B.
- Eden Model (2+1 dimensions) - Three-dimensional system growing as a two dimensional surface. Figure 5B.
- Eden Model (2 dimensional unbounded domain) - Two-dimensional system with unbounded growth. Figure 6 and Figure 7.
- Eden-Diffusion (bounded domain) - Eden model with diffusive motion for a bounded system. Figure 3, Figure 4, Figure 5A.

The use of five different models may seem to be problematic, but it highlights one of the features of this type of fractal growth. All of these models are governed by the same underlying equation, the Kardar-Parisi-Zhang Equation [9], and thus all have the same limiting behaviour when analyzing the most-recent ancestor.

References

1. Cheeseman BL, Zhang D, Binder BJ, Newgreen DF, Landman KA. Cell lineage tracing in the developing enteric nervous system: superstars revealed by experiment and simulation. Journal of The Royal Society Interface. 2014;11(93):20130815.

2. Eden M. A two-dimensional growth process. Proceedings of the Fourth Berkeley Symposium on Mathematical Statistics and Probability, Volume IV: Biology and Problems of Health, edited by J Neyman (University of California Press, Berkeley). 1961:223-239.

3. Wolf DE, Kertész J. Noise reduction in Eden models. I. Journal of Physics A: Mathematical and General. 1987;20(4):L257-L261.

4. Ohgiwari M, Matsushita M, Matsuyama T. Morphological changes in growth phenomena of bacterial colony patterns. Journal of the Physical Society of Japan. 1992;61(3):816-822.

5. Ausloos M, Vandewalle N, Cloots R. Magnetic Eden Model. EPL (Europhysics Letters). 1993;24(8):629-634.

6. Ivanenko YV, Lebovka NI, Vygornitskii NV. Eden growth model for aggregation of charged particles. The European Physical Journal B - Condensed Matter and Complex Systems. 1999;11(3):469-480.

7. Cieplak M, Maritan A, Banavar JR. Invasion percolation and Eden growth: Geometry and universality. Physical review letters. 1996;76(20):3754.

8. Yu J, Amar JG. Dynamical scaling behavior in two-dimensional ballistic deposition with shadowing. Physical Review E. 2001;66(2):021603.

9. Kardar M, Parisi G, Zhang YC. Dynamic scaling of growing interfaces. Physical Review Letters. 1986;56(9):889.

Video Description Supplement

Two supplementary videos are included. The videos were included in order to:

- Illustrate visually the function of the Cheeseman and tumour or colony growth models.
- Demonstrate clearly how the generation (time) and spatial features of the growth models are related.
- And to visually demonstrate what is meant by lineage and most recent common ancestor (MRCA) in the cell lineage study.

A description of each video follows.

Cheeseman.mp4 Video Description

This video is split by the time in which events occur.

(0:00 - 0:27) - Demonstration of Cheeseman growth. Generations 1 (red), 25 (yellow), 50 (green), 75 (blue), and 100 (magenta) are marked out for clarity. Things of note: (1) The diffusive action of the cells are evident as the colony is no longer connected and individual cells can wander away from the body of the colony. (2) The cells themselves, however, eventually become locked in at a specific position with progressively smaller probability of positional change. This is especially clear with the coloured generations where initial diffusion can be seen on the boundary growth layer, but eventually all movement ceases away from the boundary.

(0:28 - 0:39) - Spatial structure of cell generations. The marked generations (1 (red), 25 (yellow), 50 (green), 75 (blue), and 100 (magenta)) are now overlaid with an ensemble distribution. This distribution is comprised of 10000 instances of the simulation with each lattice point having a transparency value proportional to the likelihood of containing a cell in that generation. Things of note: (1) The spatial structure of the growing domain has generations separated into distinct bands with a growing variance. (2) The diffusive nature of the model allows for large deviations from expected behaviour (For example, the origin cell (red) ultimately moves 8 steps from its original position), but the generations tend to be completely contained within the expected distribution.

(0:40 - 0:56) - Most recent common ancestor (MRCA) determination. In this second part of the video the MRCA is marked out in black, the current generation of concern is in green, and the active lineages going back in time from this generation are in red. The lineage path is also marked with black lines with blue dashed lines utilized when lineages cross the periodic boundary layer for. Note that the green cells comprise all cells of a certain generation (the current generation number is shown in the top left in green text), and that the study is performed after all cells are locked into their final positions (not during actual growth). The MRCA is given as the number of the first generation in the past where only one active lineage can be found. Things of note: (1) There is a wide range of cell count numbers for the generations (the number of green cells), something not considered with basic Wright-Fisher growth. (2) The average MRCA is around 12 generations in the past, reflected in a maximum MRCA of 29 generations backwards in time shown in the top right.

Combining all of the results it should be clearer how knowledge of a type of growth (e.g. boundary growth, common in systems where either space or nutrients are at a premium) could be used to determine not only the generation (a reflection of time) in which a common ancestor can be found, but how this will offer the possibility of locating the spatial position where a common ancestor might be found. Ultimately it is clear that the Superstars found in the study by Cheeseman can be considered to be an inevitable result of lineage coalescence.

Tumour.mp4 description

This video has much the same structure of Cheeseman.mp4 and so some descriptions are reused.

(0:00 - 0:28) - Demonstration of tumour or colony growth. Generations 1 (red), 25 (yellow), 50 (green), 75 (blue), and 100 (magenta) are marked out for clarity from 40000 simulated cells. Things of note: (1) Here diffusion of the cells are not modelled and thus a clearly connected colony is evident. (2) Thus, the cells are inherently locked into their original growth positions. This also means there is a maximum distance from the origin a certain generation can exist (the 100th generation can be at most 100 steps from the origin).

(0:29 - 0:44) - Spatial segregation of generations. The marked generations (1 (red), 25 (yellow), 50 (green), 75 (blue), and 100 (magenta)) are now overlaid with an ensemble distribution. This distribution is comprised of 10000 instances of the simulation with each lattice point having a transparency value proportional to the likelihood of containing a cell in that generation. Things to note: (1) Again, the generations separate themselves into distinct spatially segregated bands. Thus, a cell of generation 100 has a well characterized expected distance from the origin in such a growth system. (2) Without diffusion the cells are now more obviously contained within the expected spatial distribution than if diffusion is possible. The effect of diffusion is something that must be considered in future studies where analysis of real world data is a possibility.

(0:44 - 0:59) - Most recent common ancestor and lineage tracing in tumour growth. Here the system is expanded such that over 400,000 cells have been simulated with over 500 generations now complete. The MRCA is marked in black and the current generation of study marked in green. The current generation number is shown in the top left, and the MRCA in the top right. The active lineages are in red. Things to note: (1) The star-shaped lineage, where the MRCA is always the origin for the entire 500 generation simulation. This is a well-known result in coalescent theory for growing populations. (2) As can be seen, the number of cells in a generation is proportional to the circumference of the boundary layer, and thus the generational population grows linearly forming a star-like lineage.

The tumour growth model is an unbounded version of the Eden growth model (with growing population size). There is no true MRCA (it is always at or near the origin), but the number of active lineages can be studied using the same fractal and coalescent theoretic methods.
